# MicroRNA-340-5p modulates cisplatin resistance by targeting LPAATβ in osteosarcoma

**DOI:** 10.1590/1414-431X20176359

**Published:** 2017-04-20

**Authors:** L. Song, P. Duan, Y. Gan, P. Li, C. Zhao, J. Xu, Z. Zhang, Q. Zhou

**Affiliations:** 1 Department of Orthopedics, First Affiliated Hospital, Third Military Medical University, Chongqing, China; 2 Southwest Eye Hospital, First Affiliated Hospital, Third Military Medical University, Chongqing, China

**Keywords:** Sensitivity to cisplatin, LPAATβ, miR-340-5p, Osteosarcoma

## Abstract

MicroRNAs (miRNAs) play an important role in drug resistance and modulate the efficiency of chemotherapy. A recent study indicated that miR-340 functions as a tumor suppressor in various types of cancer. However, the role of miR-340 in chemotherapy has not been reported yet. In this study, we found that miR-340 enhanced cisplatin (CDDP)-induced cell death. Induction of miR-340-5p expression decreased the IC_50_ of CDDP and increased the apoptosis of CDDP-resistant MG-63 and Saos-2 cells. Moreover, miR-340-5p decreased the accumulation of MRP1 and MDR1. We further explored the mechanism underlying the promoting effects of miR-340-5p on CDDP-induced cell death. We identified a potential target of miR-340 in the 3′ untranslated region of lysophosphatidic acid acyltransferase (LPAATβ) using the online program Targetscan (http://www.microrna.org). Luciferase reporter assays showed that miR-340 binds to the 3′UTR of LPAATβ. Enforced expression of miR-340-5p decreased the accumulation of LPAATβ in both MG-63 and Saos-2 cells. Silencing LPAATβ decreased the IC_50_ of CDDP and increased the apoptosis of CDDP-resistant MG-63 and Saos-2 cells, which is consistent with the effect of miR-340-5p on CDDP-induced cell death. Moreover, induced expression of LPAATβ compromised the effects of miR-340-5p on CDDP-induced cell death and accumulation of MRP1 and MDR1. Taken together, our data indicated that miR-340-5p enhanced the sensitivity to CDDP by targeting LPAATβ.

## Introduction

Osteosarcoma (OS) is an aggressive malignant neoplasm that arises from primitive transformed cells of mesenchymal origin, exhibits osteoblastic differentiation, and produces malignant osteoid. OS is ranked highest in morbidity among all primitive malignant tumors (1). Although chemotherapy is frequently used in OS, many factors lead to its failure. Drug resistance is the main factor affecting the efficiency of chemotherapy (1). Previous studies indicated that drug resistance is a complicated process involving many genes, including microRNAs (miRNAs) (2–4).

miRNAs are non-coding RNAs approximately 18-22 nucleotides in length (5). Recent studies demonstrated that miRNAs are key regulators of tumor initiation and progression (6–8). They typically modulate proliferation, migration, invasion and drug resistance of tumor cells by targeting oncogenes, tumor suppressor genes, transcription factors, and other regulators involved in cell death and survival (7,9–11). MicroRNA-340 (miR-340) was first reported to be a suppressive miRNA in breast cancer (12). Subsequent studies of colorectal cancer (13), osteosarcoma (14), melanoma (15), and gastric cancer (16) confirmed that miR-340 plays an inhibitory role in the proliferation, migration, and invasion of cancer cells.

1-Acylglycerol-3-phosphate *O-*acyltransferase 2, also known as lysophosphatidic acid acyltransferaseβ (LPAATβ), is a member of the 1-acylglycerol-3-phosphate *O*-acyltransferase family (17). The protein is located within the endoplasmic reticulum membrane and converts lysophosphatidic acid to phosphatidic acid, the second step in *de novo* phospholipid biosynthesis (17). Recent studies in ovarian cancer suggested that LPAATβ plays a role in tumor progression (18,19). Rastegar et al. (20) reported that LPAATβ promotes the tumor growth of human OS. It also functions as a downstream target of miRNA (21). MicroRNA-24 inhibits cell proliferation by targeting LPAATβ in OS (21). LPAATβ is also involved in drug resistance (22). The LPAATβ inhibitor CT-32615 triggers caspase-dependent apoptosis and can overcome resistance to conventional therapeutics (i.e., dexamethasone, doxorubicin, melphalan) in multiple myeloma cells (22).

In the current study, we analyzed the expression of miR-340-5p in OS and CDDP-resistant cells and examined the effects of miR-340-5p on CDDP-induced cell death and expression of drug resistance-related genes. We also investigated the mechanism underlying the transcription regulation of miR-340-5p on LPAATβ. Our results provide novel insight into the CDDP resistance of OS, which may help to improve the efficacy of chemotherapy.

## Material and Methods

### Cell culture

OS cell lines MG-63 and Saos-2, and CDDP-resistant OS cells MG-63/CDDP and Saos-2/CDDP were obtained from Shanghai Cell Institute (China). MG-63, Saos-2,MG-63/CDDP, and Saos-2/CDDP cells were grown in 1640 medium containing 10% fetal bovine serum (Gibco/BRL, USA) supplemented with 100 U/mL penicillin G and 100 μg/mL streptomycin (Sigma-Aldrich, USA). Cells were maintained at 37°C in a humidified 5% CO_2_ incubator.

### Constructs

To create the luciferase reporter constructs containing the wild-type 3′ untranslated region of LPAATβ, the full-length 3′UTR of LPAATβ was amplified and cloned into the pmirGLO vector (Promega, USA). The primers used to amplify the 3′UTR of LPAATβ are as follows: forward 5′CTAGGCATGCAGACCACGGCAGGGCATG3′ and reverse 5′CCCAAGCTTTTGCCACTTCCAAGAGTGTG3′. Luciferase reporter constructs containing mutated binding sites were created using the QuikChange¯ Site-Directed Mutagenesis Kit (Strategene, USA) with the wild-type 3′UTR of LPAAT β as a template. The primers used to amplify the 3′UTR of LPAATβ were as follows: 5′CACTGTACTCCGTTGCTGTTTTTATCTGAACACACTCTTGGAAGTGGC3′ and 5′GCCACTTCCAAGAGTGTGTTCAGATAAAAACAGCAACGGAGTACAGTG3′. The LPAATβ expression constructs were created by subcloning the coding region of LPAATβ into a pcDNA3.0-expressing vector (Invitrogen, USA). The primers used to amplify the 3′UTR of LPAATβ were as follows: 5′CGGGGTACCATGGAGCTGTGGCCGTGTC3′ and 5′GCCACTTCCAAGAGTGTGTTCAGATAAAAACAGCAACGGAGTACAGTG3′. All constructs were verified by sequencing.

### Transfection of miRNAs and siRNAs

The mimics of miR-340-5p and siRNAs targeted to LPAATβ were obtained from GenePharma Co., Ltd. (China). First, 2×10^4^ OS cells were plated into 6-well plates the day before transfection. Next, 100 nM miRNAs or siRNAs were transfected into OS cells using Lipofectamine 2000 reagent (Invitrogen) according to the manufacturers’ instruction. A scramble sequence was used as the negative control (NC). The transfection efficiency of miRNAs and siRNAs was determined by quantitative real-time RT-PCR (qRT-PCR) and western blot analysis, respectively.

### qRT-PCR

Total RNA was extracted using TRIZOL reagent (Ambion, USA) according to the manufacturer’s instructions. cDNA used to examine the expression of LPAATβ was synthesized using the PrimeScript™ RT reagent kit (TaKaRa, Japan) according to the manufacturer’s instructions. Expression of LPAATβ was examined using SYBR¯ Premix Ex Taq™ II (TaKaRa) and GAPDH served as internal reference. All experiments were performed in duplicate and repeated twice. The results are represented as the fold-induction using the 2^-ΔΔCT^ method. Primers used to examine the expression of LPAATβ were as follows: LPAATβ, forward: 5′-CCTTCCTCCACATCTCCAAG-3′, reverse: 5′-CCGGACAGAGTGGTATTTGG-3′; miR-340-5p, forward: 5′-GCGGTTATAAAGCAATGAGA-3′, reverse: 5′-GTGCGTGTCGTGGAGTCG-3′; U6, forward: 5′-CTCGCTTCGGCAGCACA-3′, reverse: 5′-AACGCTTCACGAATTTGCGT-3′.

### Western blot analysis

Western blot analysis was performed according to standard procedures as previously described (23). Briefly, proteins were separated by 10% SDS-PAGE and then transferred to nitrocellulose membranes (Bio-Rad, USA). After blocking in 5% nonfat milk, the membranes were incubated with the following primary antibodies: rabbit anti-LPAATβ polyclonal antibody (ab62599; 1:500; Abcam, UK), rabbit anti-MRP1 polyclonal antibody (ab180960; 1:500; Abcam), rabbit anti-MDR1 polyclonal antibody (ab170904; 1:500; Abcam), and rabbit anti-GAPDH mAb (1:1,000; Abcam). The proteins were visualized using enhanced chemiluminescence reagents (Pierce, USA).

### Proliferation assays

Cell Counting Kit-8 (CCK8) was used to evaluate the growth of OS cells treated with CDDP according to the manufacturer’s protocol. Briefly, 10^4^ cells/well were plated in triplicate in 96-well plates. CCK8 solution was added to each well at a 1:10 dilution. Cells were incubated for 4 h, and then the absorbance at 450 nm was measured using a multi-well plate reader. The IC50 value was calculated using SPSS software (USA).

### Apoptosis assays

An Annexin V-FITC apoptosis detection kit (Multisciences, China) was used to detect apoptosis in OS cells. According to the manufacturer’s instructions, the cells were digested with trypsin and centrifuged at 300 *g* for 5 min at 4°C. After collection, the cells were washed twice with PBS and centrifuged at 300 *g* for 5 min at 4°C, and 3×10^5^ cells were collected and suspended in 500 μL binding buffer. Next, 5 μL Anexin V-FITC and 5 μL propidium iodide were added and mixed at room temperature in the dark for 15 min. Within 1 h, the cells were detected by flow cytometry.

### Luciferase reporter assays

OS cells were seeded onto 96-well plates at 6000 cells per well the day before transfection. A mixture of 100 ng luciferase reporter constructs (pmirGLO-LPAATβ-WT and pmirGLO-LPAATβ-mutant) and 200 ng of NC or miR-340-5p mimics was transfected into OS cells with Lipofectamine 2000. Forty-eight hours later, Firefly and Renilla luciferase activities were measured using a Dual-Luciferase Reporter System (Promega) according to the manufacturer’s protocol.

### Statistical analysis

Data are reported as means±SD, unless otherwise indicated. Student’s *t*-test was used to analyze statistical differences between groups. A P value <0.05 was considered to be statistically significant.

## Results

### MiR-340-5p was down-regulated in CDDP-resistant OS cells

**Figure 1:**
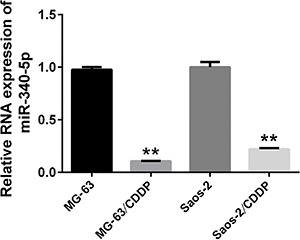
miR-340-5p is down-regulated in cisplatin (CDDP)-resistant osteosarcoma cells. The expression of miR-340-5p was examined in MG-63, Saos-2, MG-63/CDDP, and Saos-2/CDDP cells. U6 served as an internal reference. The relative expression of miR-340-5p was calculated using the 2^-ΔΔCT^ method. All experiments were repeated three times. Data are reported as means±SD. **P<0.05 compared to its respective control (Student’s *t*-test).

In order to determine the role of miR-340-5p in CDDP resistance, we first examined the expression profile of miR-340-5p in CDDP-resistant OS cells, MG-63/CDDP and Saos-2/CDDP. Normal OS cells, MG-63 and Saos-2, were used as controls. qRT-PCR was performed to examine the expression of miR-340-5p. Our data showed that the expression of miR-340-5p in CDDP-resistant OS cells was much lower than in OS cells (Figure 1). This suggests that miR-340-5p was down-regulated in CDDP-resistant OS cells.

### Effect of miR-340-5p on CDDP resistance in OS cells

**Figure 2:**
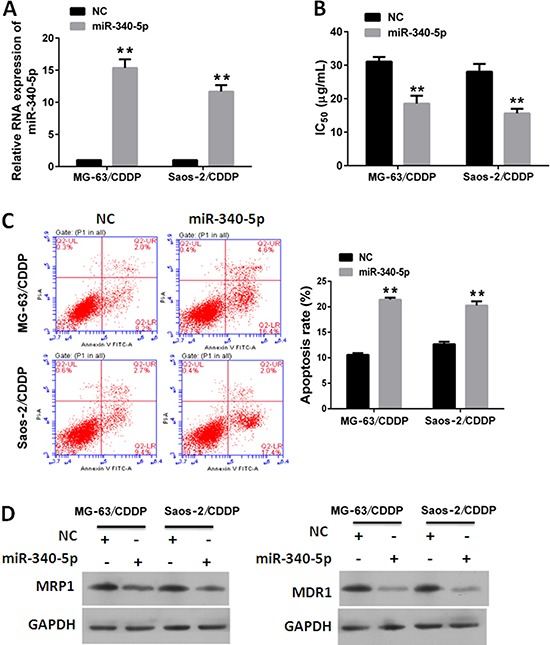
miR-340-5p enhanced sensitivity of cisplatin (CDDP) in osteosarcoma cells. *A*, Relative RNA expression of miR-340-5p in MG-63/CDDP and Saos-2/CDDP cells transfected with miR-340-5p or normal control (NC). *B*, miR-340-5p decreased IC_50_ of CDDP in MG-63/CDDP and Saos-2/CDDP cells. *C*, miR-340-5p increased CDDP-induced apoptosis in MG-63/CDDP and Saos-2/CDDP cells. Data are reported as means±SD. **P<0.01 (Student’s *t*-test). *D*, miR-340-5p decreased the expression of MRP1 and MDR1 in MG-63/CDDP and Saos-2/CDDP cells exposed to CDDP.

MG-63/CDDP and Saos-2/CDDP cells were treated with CDDP (15 µg/mL) following transfection of miR-340-5p mimics. A scramble RNA sequence was used as a NC. The results of qRT-PCR showed that miR-340-5p was successfully overexpressed after transfection of miR-340-5p mimics compared to NC (Figure 2A). The IC_50_ of CDDP was calculated based on the CCK8 assays. The IC_50_ values of CDDP in MG-63/CDDP and Saos-2/CDDP cells transfected with miR-340-5p were lower than those transfected with NC (Figure 2B).

We then investigated the effects of miR-340-5p on CDDP-induced apoptosis in MG-63/CDDP and Saos-2/CDDP cells. Our data showed that the apoptosis rates in cells transfected with miR-340-5p were higher than in those transfected with NC (Figure 2C).

We also investigated the effects of miR-340-5p on drug transporters, including MRP1 and MDR1 (24–26). Our data showed that the accumulations of MRP1 and MDR1 in cells transfected with miR-340-5p were lower than in those transfected with NC (Figure 2D). These results suggest that miR-340-5p impaired CDDP resistance in OS cells.

### MiR-340-5p down-regulated LPAATβ

**Figure 3:**
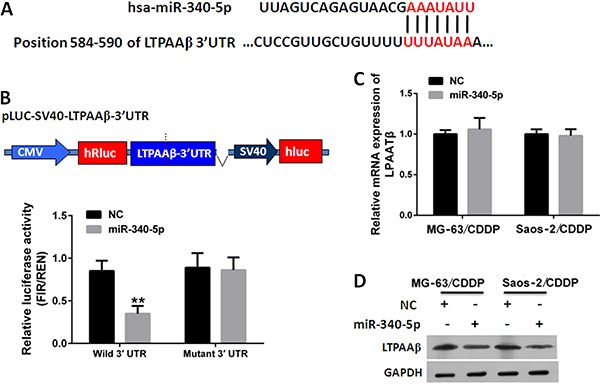
*A*, Schematic diagram of interaction between miR-340-5p and LPAATβ. *B*, Dual luciferase assay of cells co-transfected with luciferase reporter constructs and miR-340-5p or normal control (NC). *C*, miR-340-5p did not affect mRNA expression of LPAATβ in MG-63/CDDP and Saos-2/CDDP cells. Data are reported as means±SD. **P<0.01 (Student’s *t*-test). *D*, miR-340-5p decreased protein expression of LPAATβ in MG-63/CDDP and Saos-2/CDDP cells.

In order to explore the mechanism underlying the inhibitory effects of miR-340-5p on CDDP resistance, we identified the target of miR-340-5p using an online program (http://www.microrna.org). LPAATβ showed high scores and a miR-340-5p binding site in its 3′UTR (Figure 3A). In order to investigate whether LPAATβ is a target of miR-340-5p, we created luciferase reporter constructs containing wild-type or mutated 3′UTRs of LPAATβ, and examined whether miR-340-5p binds to the 3′UTR of LPAATβ.

Our data showed that miR-340-5p expression was decreased relative to luciferase activities in MG-63 cells transfected with pmirGLO-LPAATβ-WT, but not in MG-63 cells transfected with the pmirGLO-LPAATβ-mutant (Figure 3B). These results indicate that miR-340-5p binds to the 3′UTR of LPAATβ.

Next, we examined the mRNA and protein expression of LPAATβ in MG-63/CDDP and Saos-2/CDDP cells transfected with mimics of miR-340-5p. Our data showed that expression of miR-340-5p did not affect the mRNA expression of LPAATβ (Figure 3C). However, protein accumulation of LPAATβ was decreased in MG-63/CDDP and Saos-2/CDDP cells (Figure 3D), indicating that miR-340-5p acts as a negative regulator of LPAATβ.

**Figure 4:**
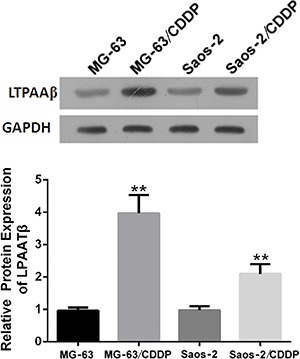
LPAATβ was up-regulated in cisplatin (CDDP)-resistant osteosarcoma cells. The expression of LPAATβ was examined in MG-63, Saos-2, MG-63/CDDP, and Saos-2/CDDP cells using western blot analysis. GAPDH served as loading control. Upper: representative image of western blot analysis. Lower: histogram represents the densitometry data for at least 3 independent experiments. Data are reported as means±SD. **P<0.01, compared to its respective control (Student’s *t*-test).

To further verify our previous finding, we investigate the expression of LPAATβ in CDDP-resistant OS cells, MG-63/CDDP and Saos-2/CDDP, in which miR-340-5p proved to be down-regulated. Normal OS cells, MG-63 and Saos-2, were used as controls. Western blot analysis showed that the expression of LPAATβ in CDDP-resistant OS cells was much higher than in OS cells (Figure 4). These data suggested that LPAATβ was up-regulated in MG-63/CDDP and Saos-2/CDDP cells, in which miR-340-5p proved to be down-regulated. This result partially supports our previous finding that miR-340-5p acted as a negative regulator of LPAATβ.

### Effect of silencing LPAATβ on CDDP resistance

**Figure 5:**
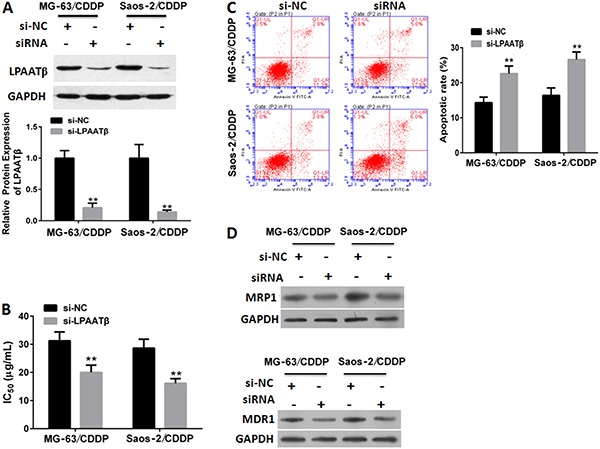
Effect of silencing LPAATβ on cisplatin (CDDP) resistance. *A*, Relative protein expression of LPAATβ in MG-63/CDDP and Saos-2/CDDP cells transfected with siRNA or normal control (NC). *B*, Silencing LPAATβ decreased IC_50_ of CDDP in MG-63/CDDP and Saos-2/CDDP cells. *C*, Silencing LPAATβ increased CDDP-induced apoptosis in MG-63/CDDP and Saos-2/CDDP cells. Data are reported as means±SD. **P<0.01, compared to its respective control (Student’s *t*-test). *D*, Silencing LPAATβ decreased the expression of MRP1 and MDR1 in MG-63/CDDP and Saos-2/CDDP cells exposed to CDDP.

We demonstrated that LPAATβ is a target of miR-340-5p. In order to verify that miR-340-5p modulated CDDP resistance by down-regulating LPAATβ, we investigated the effects of silencing LPAATβ on CDDP resistance. If effects of silencing LPAATβ were consistent with effects of miR-340-5p, it would be partially proven that miR-340-5p modulated CDDP resistance targeting LPAATβ. The siRNAs targeted LPAATβ were used to suppress LPAATβ in MG-63/CDDP and Saos-2/CDDP cells. A scramble RNA was used as NC. The silencing efficiency of siRNA was confirmed by decreased accumulation of LPAATβ in cells transfected with siRNAs (Figure 5A). MG-63/CDDP and Saos-2/CDDP cells were transfected with siRNAs or NC and treated with CDDP for 24 hours. IC_50_ value was determined using CCK8 assays. Our data showed IC50 value of siRNA group was lower than those of NC group (Figure 5B). The effects of silencing LPAATβ on CDDP induced-apoptosis was determined by flow cytometry. The results showed the apoptosis rate of siRNA group was higher than those of the NC group (Figure 5C). These results indicate that the effects of silencing LPAATβ on CDDP resistance were consistent with those of miR-340-5p.

### Enforced expression of LPAATβ attenuated effects of miR-340-5p on CDDP resistance

**Figure 6:**
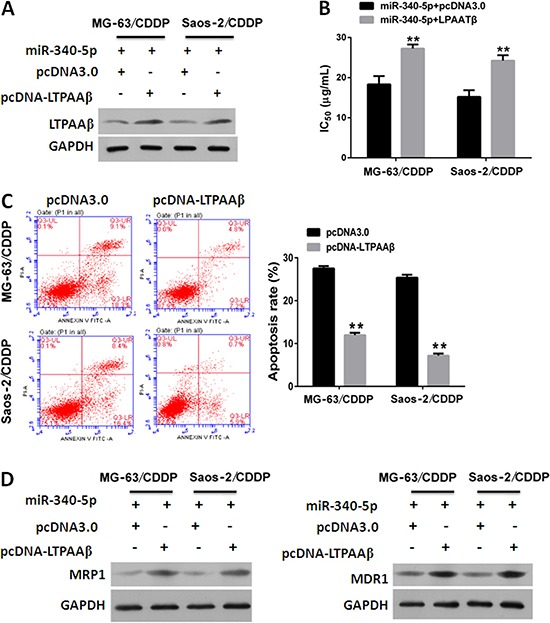
Expression of LPAATβ attenuated inhibitory effects of miR-340-5p on cisplatin (CDDP) resistance. *A*, Expression of LPAATβ in MG-63/CDDP and Saos-2/CDDP cells co-transfected with miR-340-5p and pcDNA-LPAATβ or pcDNA3.0. *B*, Expression of LPAATβ compromised inhibitory effects of miR-340-5p on IC50 of CDDP in MG-63/CDDP and Saos-2/CDDP cells. *C*, Expression of LPAATβ attenuated promoting effects of miR-340-5p on CDDP-induced apoptosis in MG-63/CDDP and Saos-2/CDDP cells. Data are reported as means±SD. **P<0.01 (Student’s *t*-test). *D*, Expression of LPAATβ compromised inhibitory effects of miR-340-5p on expression of MRP1 and MDR1 in MG-63/CDDP and Saos-2/CDDP cells exposed to CDDP.

We demonstrated that LPAATβ is a target of miR-340-5p. In order to verify that miR-340-5p modulates CDDP resistance by down-regulating LPAATβ, we evaluated whether up-regulation of LPAATβ affected the role of miR-340-5p in CDDP resistance. We co-transfected expression plasmids of pcDNA-LPAATβ and miR-340-5p into MG-63/CDDP and Saos-2/CDDP cells. The control group was transfected with the pcDNA3.0 vector and miR-340-5p. Transfection efficiency was confirmed by over-expression of LPAATβ in cells transfected with pcDNA-LPAATβ (Figure 6A). We found that the IC_50_ of CDDP in cells transfected with pcDNA-LPAATβ was higher than in those transfected with pcDNA3.0 (Figure 6B), suggesting that expression of LPAATβ attenuated the effect of miR-340-5p on the IC_50_ of CDDP.

We also investigated whether expression of LPAATβ affected the inhibitory effect of miR-340-5p on CDDP-induced apoptosis. Our data showed that apoptosis in the pcDNA-LPAATβ group was lower than that in the control group (Figure 6C), suggesting that LPAATβ expression alleviated the promoting effect of miR-340-5p on CDDP-induced apoptosis. In addition, we found that expression of MRP1 and MDR1 in the pcDNA-LPAATβ group was higher than in the control group (Figure 6D). Taken together, these results suggest that expression of LPAATβ attenuated the effects of miR-340-5p on CDDP resistance.

## Discussion

Chemotherapy is an effective treatment for OS. However, many patients develop primary and secondary drug resistance, causing chemotherapy to fail. It is very important to enhance the sensitivity of OS cells to chemotherapy reagents. However, the molecular mechanism of chemotherapy resistance is not fully understood. Recent studies revealed numerous genes or noncoding RNA molecules involved in the regulation of sensitivity to chemotherapy reagents. In the current study, we found that miR-340 plays an important role in enhancing the sensitivity of OS cells to CDDP.

MiR-340 was first reported in melanoma as a regulator of microphthalmia-associated transcription factor (MITF) (27). Recent studies of breast cancer (12,28,29), colorectal cancer (13,30), melanoma (15), gastric cancer (16), glioblastoma (31,32), hepatocellular carcinoma (33), lung cancer (34), oral squamous cell carcinoma (35), and laryngeal squamous cell carcinoma (36) indicated that miR-340 is involved in the proliferation, metastasis, invasion, and apoptosis of cancer cells. Other studies demonstrated that miR-340 serves as a negative regulator in cancer (12,31,37–39). Zhou et al. (40) reported that miR-340 suppresses tumor growth and metastasis in OS. The expression of miR-340 has been associated with tumor progression and prognosis in pediatric OS (14). Our data indicates that miR-340 regulates CDDP-induced apoptosis in CDDP-resistant OS cells, suggested that miR-340 enhanced the sensitivity of OS cells to CDDP.

The ATP-binding cassette (ABC) transporters transport various molecules across extra- and intra-cellular membranes (24). They had been proven by various studies to modulate the development of resistance to anticancer drugs (24). MRP1 (ABCC1) is a member of the MRP subfamily, which is involved in multi-drug resistance (25). This protein functions as a multispecific organic anion transporter, with oxidized glutathione, cysteinyl leukotrienes, and activated aflatoxin B1 as substrates (25). This protein also transports glucuronides and sulfate conjugates of steroid hormones and bile salts (25). MDR1 (ABCB1) is a member of the MDR/TAP subfamily. Members of the MDR/TAP subfamily are involved in multidrug resistance. MDR1 is an ATP-dependent drug efflux pump for xenobiotic compounds with broad substrate specificity (26). It is responsible for decreased drug accumulation in multidrug-resistant cells and often mediates the development of resistance to anticancer drugs (26). It also functions as a transporter in the blood-brain barrier (26). Our data showed that miR-340 decreased MRP1 and MDR1 expression. These data support our previous finding that miR-340 modulated CDDP resistance in OS cells.

MiRNAs typically accomplish their function by modulating the expression of their target genes. Identifying target genes is crucial for understanding the role of miRNAs in cancer cells. We identified LPAATβ as a novel target of miR-340 in OS. Several genes have been identified as targets of miR-340, including MITF (23), c-Met (12), ROCK1 (36), RhoA (37), Nrf2 (29), p27 (33), MYO10 (34), NRAS (28), MDM2 (35), NF-x03BA, and CTNNB1 (25). MiR-340 binds to the 3′UTR of target genes and decreases the accumulation of target genes in cancer cells (12,23,28,30,33,36,38). Our findings are consistent with those of previous studies. We showed that miR-340 binds to the 3′UTR of LPAATβ. Mutation in the biding site of miR-340 compromised its binding to the 3′UTR of LPAATβ. In addition, we found that the effects of LPAATβ knockdown on CDDP resistance are similar to the effects of miR-340-5p on CDDP resistance in both MG-63/CDDP and Saos-2/CDDP cells. We also demonstrated that over-expression of LPAATβ attenuated the effects of miR-340-5p on CDDP resistance. These results indicate miR-340 affected CDDP resistance by down-regulating LPAATβ.

In conclusion, we found that miR-340 enhanced the sensitivity of OS to CDDP by targeting LPAATβ. Our study provides insight into CDDP resistance in OS. MiR-340 may serve as a target for chemotherapy of OS. However, there were several limitations to our study. First, *in vivo* evidence of the role of miR-340 in chemotherapy is required to support the findings of our study. Second, up-stream regulators of the miR-340-LPAATβ axis are unclear, which affects application of the miR-340-LPAATβ axis in chemotherapy. Third, down-stream signaling pathways of miR-340-LPAATβ axis have not been determined.
